# A food web including parasites for kelp forests of the Santa Barbara Channel, California

**DOI:** 10.1038/s41597-021-00880-4

**Published:** 2021-04-08

**Authors:** Dana N. Morton, Cristiana Y. Antonino, Farallon J. Broughton, Lauren N. Dykman, Armand M. Kuris, Kevin D. Lafferty

**Affiliations:** 1 Department of Ecology, Evolution, and Marine Biology, University of California, Santa Barbara, CA 93106-6150 USA; 2 Marine Science Institute, University of California, Santa Barbara, CA 93106-9610 USA; 3 College of Creative Studies, University of California, Santa Barbara, CA 93106-6150 USA; 4 Western Ecological Research Center, U.S. Geological Survey, at Marine Science Institute, University of California, Santa Barbara, CA 93106-9610 USA

**Keywords:** Food webs, Marine biology, Ecological networks

## Abstract

We built a high-resolution topological food web for the kelp forests of the Santa Barbara Channel, California, USA that includes parasites and significantly improves resolution compared to previous webs. The 1,098 nodes and 21,956 links in the web describe an economically, socially, and ecologically vital system. Nodes are broken into life-stages, with 549 free-living life-stages (492 species from 21 Phyla) and 549 parasitic life-stages (450 species from 10 Phyla). Links represent three kinds of trophic interactions, with 9,352 predator-prey links, 2,733 parasite-host links and 9,871 predator-parasite links. All decisions for including nodes and links are documented, and extensive metadata in the node list allows users to filter the node list to suit their research questions. The kelp-forest food web is more species-rich than any other published food web with parasites, and it has the largest proportion of parasites. Our food web may be used to predict how kelp forests may respond to change, will advance our understanding of parasites in ecosystems, and fosters development of theory that incorporates large networks.

## Abstract (summary)

**Table:** 

Measurement(s)	Host-Parasite Relationship • food web • ecological community
Technology Type(s)	Dissection • digestive tract environment • digital curation
Sample Characteristic - Organism	Vertebrata • Platyhelminthes • Arthropoda • Mollusca • Macrocystis pyrifera
Sample Characteristic - Environment	kelp forest
Sample Characteristic - Location	Santa Barbara Channel

Machine-accessible metadata file describing the reported data: 10.6084/m9.figshare.13487178

## Background & Summary

**Fig. 1:**
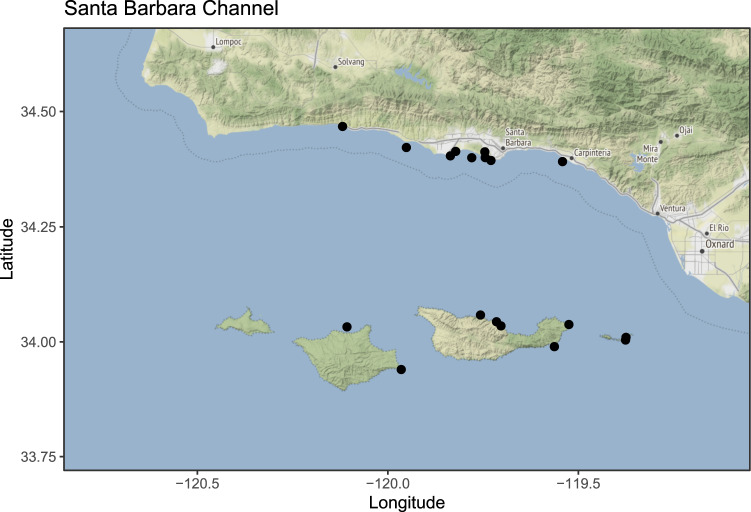
The study region and sampling sites. The study region extends from Point Conception to Point Mugu and includes the four islands that delineate the Santa Barbara Channel (from east to west: Anacapa, Santa Cruz, Santa Rosa, San Miguel). Black dots indicate sites where sampling for parasites occurred^30^. Map citation: Google Maps™ via *ggmap* in R^52^.

Parasites are ubiquitous in food webs^1^, but only a few food webs systematically include parasites^2,3^. Parasites can have strong effects on diversity, biomass, and food-web complexity^2,4–6^. Kelp-forest ecosystems are more complex, dynamic, and open than many ecosystems for which food webs with parasites have been built (salt marsh^4,6,7^, sand flat^2^, and lake ecosystems^8^). The extensive knowledge base and research history at the research site in southern California provided both the necessary foundation for this work and the motivation to build a well-resolved food web. Kelp forests along the coast of southern California (San Diego to Point Conception) have been studied more than anywhere else in the world, with over seven decades of research on feeding interactions^9–14^ and the cascading indirect effects^15–17^ that permeate the kelp-forest food web. Santa Barbara Channel (SBC) kelp forests (Fig. 1) were ideal for this work due to monitoring by the Channel Islands National Park and the SBC Long Term Ecological Research programs.

Previous studies have constructed food webs for kelp forests, but these lack resolution that would allow for network analysis, are difficult to compare with other food webs due to the methodology and structure, and they do not include parasites. Three food webs and a links database have been published for California kelp forests^18–21^. The nodes in these webs are often highly aggregated for certain taxonomic groups (e.g. all amphipods combined) but resolved to the species level for other taxa (notably fishes). Many invertebrate groups are altogether missing from these webs, as these webs were largely constructed based off SCUBA diver surveys. A high-resolution food web published for intertidal rocky habitats in Chile^22^ also included non-trophic interactions but does not include small cryptic organisms, notably parasites, amphipods, isopods, fish, and many other small mobile invertebrates. Further studies have examined kelp-forest food-web structure via stable isotopes (e.g. Chile^23^, France^24^, Norway^25^, and Southern California^26–29^) and either aggregated species to functional groups, did not attempt to assign trophic interactions between consumer species, or focused on a subset of interactions within the system. We have added to this extensive knowledge base to build an improved kelp-forest food web that systematically resolves the free-living and parasitic species that dominate biodiversity in this system. The resulting food web is more species rich than any other published food web with parasites, and has a larger proportion of parasitic species than other webs (47.7% in kelp forests, 38% in tropical sand flat^2^, 30–34.8% in salt marshes^4,6,7^, 26% in lake ecosystems^8^). We compare the kelp-forest food web structure in detail with other published food webs of similar construction (salt marsh^4,6,7^, tropical sand flat^2^, and lake ecosystems^8^) in a separate manuscript in preparation.

A food web starts with a list of nodes for a given location and time period, and then follows with a determination of which of the potential feeding links among nodes occur. This process by which nodes or links are included or excluded is often unclear in published food webs, so we included metadata which provides transparency and will facilitate use by other researchers. We compiled lists of free-living and parasitic kelp-forest species from several sources through systematic literature review, augmented with extensive field sampling. Because species’ food sources and predators often change as they grow, we partitioned these species into different life stages. Therefore, most nodes in the web were resolved to species and life stage. We included metadata for justification for inclusion, confidence in node presence, locality, taxonomic relationships, and three functional traits (habitat niche, life-style, and consumer strategy) for each node^30^. These traits, in combination with the trophic relationships resolved in the food web, help define the functional roles of nodes, which make it possible for others to analyze the effects of species loss or gain, changing thermal environments, or changing habitat features (e.g. the dynamics of canopy-forming kelps). Predator-prey, parasite-host, and predator-parasite links between nodes were then obtained from published records, direct observation, expert opinion, and several types of logical inference (e.g. encounter via shared habitat, feeding behavior, compatibility via feeding method, host specificity, etc.). Link metadata includes type of trophic interaction, justification for inclusion, confidence in link presence, locality, and references^30^. This metadata will allow users to filter the node and links list according to their research questions. Taking a systematic approach increased resolution at every trophic level, leading to perhaps the most species-rich and complete marine food web yet created.

**Fig. 2:**
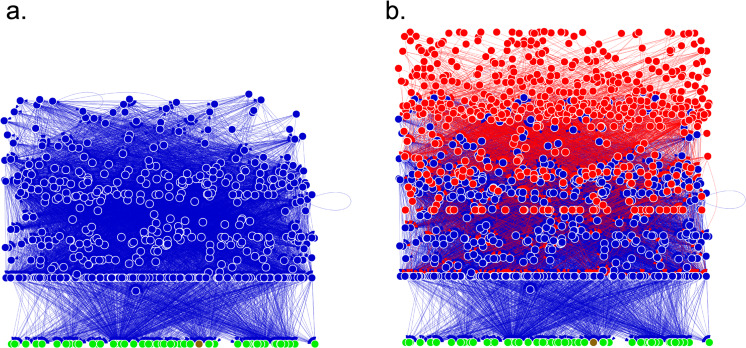
Graph of the food web without (**a**) and with (**b**) parasites, showing the magnitude of the node list, density of links in the network, and increase in network size with the inclusion of parasites. Blue nodes are free-living taxa, red nodes are parasites, green are autotrophs, and brown are detritus. Each node represents a life stage of a species, and arrows point from consumers to resources. The network is arranged vertically by prey-averaged trophic level, so top consumers are at the top and producers and detritus are at the bottom. The horizontal arrangement of nodes is random, so clusters of nodes do not represent any feature of the network. Some nodes may be obscured by other nodes due to the complexity and size of the network. Concomitant links not shown so as not to obscure the other links. Created in *igraph* in R^51^.

**Fig. 3:**
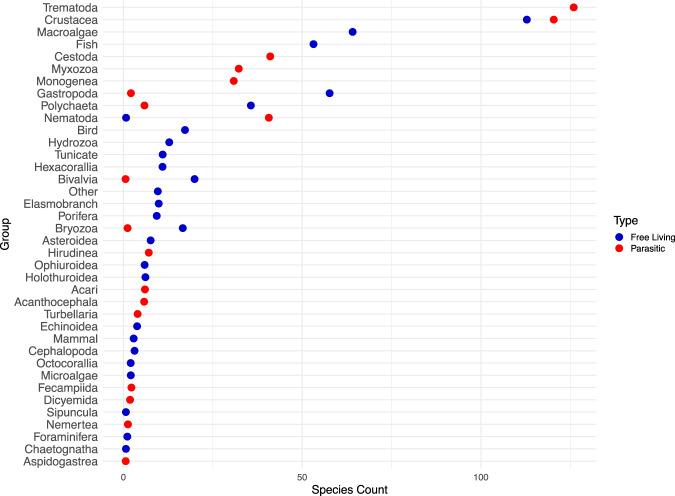
Species contributions to the food web by organismal group. Blue circles indicate the number of free-living species in that group, red indicates the number of parasites.

492 species (549 life stages) were included in the resolved free-living web (compared with 217 included in^20^). Parasites added an additional 450 species (549 life stages), comprising 47.7% of all species (Fig. 2). Improving resolution for small crustaceans and other invertebrate taxa added the most to free-living diversity compared to past efforts by others^18–21^. Platyhelminthes added the most parasitic species overall, and trematodes were the most diverse group (Fig. 3). Parasitic crustaceans (mostly copepods) were the second most species-rich group of parasites, with more parasitic crustaceans than free-living crustaceans (132 vs. 113 respectively). The predator-prey subweb comprised 42.6% of links, the predator-parasite subweb had 45.0% of links, and parasite-host subweb had 12.4% of links. The kelp-forest food web taxa became dominated by helminths and crustaceans when parasites were included.

## Methods

### Site description

We define “kelp forest” as rocky-reef habitat within the 5–20 m depth range that supports dense stands of giant kelp, *Macrocystis pyrifera*. For this study, we considered the Santa Barbara Channel (SBC) to include the mainland region between Point Conception (−120.476° longitude, 34.455° latitude) and Point Mugu (−119.065° longitude, 34.079° latitude), as well the northern and southern sides of the four northern Channel Islands (Fig. 1). Although the SBC is a subset of the Southern California Bight, its strong west-east gradient in cold to warm temperature means the study system includes many of the kelp-forest species throughout California^31^. This means the SBC kelp-forest food web is a large “metaweb”, characterizing kelp forest meta-communities, rather than a site-specific web. In other words, the system includes cold water and warm water species that might not necessarily co-occur at a single site. However, there are site-specific food webs embedded in the metaweb at particular locations where a subset of species occur.

### Data sources

Our goal was to assemble the food web using both published and novel empirical observations. To this end, we first used published data sets and species’ range boundaries to create free-living species lists. The initial list of fishes, algae, and free-living invertebrates was assembled from the Channel Islands National Park Kelp Forest Monitoring program (CINP KFM, annual reports available at https://irma.nps.gov/DataStore/SavedSearch/Profile/1508, accessed March 6, 2017, or visit https://www.nps.gov/im/medn/kelp-forest-communities.htm to contact David Kushner or Joshua Sprague) and the SBC Long Term Ecological Research program’s ongoing kelp-forest community timeseries (SBC LTER, https://sbclter.msi.ucsb.edu/data/catalog/, accessed March 12, 2017). We added to these lists using primary literature, technical reports (e.g., NOAA, USFW), direct observations, expert opinion, crowd-sourced observations (e.g., eBird.org), guidebooks, and grey literature. We sampled the local kelp forest zooplankton and the algae-associated small-invertebrate community, because these organisms were not well represented in surveys (see below).

We created initial lists of parasite species using published literature and host-parasite databases. A systematic review was conducted to collect parasite records for each free-living species. We searched the Natural History Museum (NHM) of London host-parasite database (https://www.nhm.ac.uk/research-curation/scientific-resources/taxonomy-systematics/host-parasites/database/search.jsp), the FishPest database^32^, WoRMs (http://www.marinespecies.org/aphia.php?p = search), BIOSIS citation index (http://webofscience.com), and Google Scholar™(https://scholar.google.com/) (Genus + species + parasit*, expanded to Genus + parasit* if no records were found). For each host species, we recorded the number of records found in BIOSIS and NHM as an estimate of study effort. Although parasites are often reported at the host and parasite species level, we were often able to infer parasite and host life stages based on knowledge about life cycles. We added to these lists by sampling local fish and invertebrates, with a focus on hosts that were common in the system and not well-studied (see below). As for any food-web study, we were most interested in including common or important parasites, rather than rarities.

Published diet observations (including in grey literature), direct observations, and inference were used to determine trophic links (see below).

### Free-living species sampling methods

Certain groups of free-living species were under-represented in published survey data, so we conducted sampling to assess species diversity in the following areas.

#### Zooplankton tows

We conducted vertical zooplankton tows within kelp forests at two island locations (on the same date) and two mainland locations (repeated tows, four dates at one site, three of those dates at a second site, including one nighttime sampling date), for eight site by date samples^30^. While the vessel was at anchor within a kelp forest, a 30 cm diameter, 200 micron plankton net was dropped to the bottom and pulled to the surface at a rate of 0.33 m per second. Care was taken not to scrape the net against kelp plants. The collection jar attached to the net was kept vertical with a small lead weight to ensure that the net did not collect organisms on the way down to the bottom. The depth and time of collection were recorded^30^. We held collection jars on ice while in the field, then preserved specimens in 95% ethanol when we returned to the lab (within a few hours of collection). All organisms were counted and identified to species when possible, but some groups were identified to Order or Family, and then cross-checked with lists of known local species. If this was not possible, specimens were assigned to morphospecies, indicating they appeared to be a unique species based on morphology. Representative specimens from each species or morphospecies were photographed and measured.

#### Giant kelp holdfasts

Giant kelp holdfasts were sampled for free-living invertebrates. In the field, holdfast circumference and two slant height measures were taken, as well as basal stipe circumference. A subsample of approximately 25% of the holdfast was collected by SCUBA in a large plastic zip bag, and frozen until processing (n = 7). The samples were processed for organisms > 500 microns, and holdfast tissue was weighed after organisms and debris were removed. Organisms were counted, identified to species or morphospecies when possible, and measured^30^. Some groups were identified to Family, and then matched to lists of known local species.

#### Taxon-specific methods: gastropods

Small gastropods are a diverse but overlooked group that lives in benthic turf algae. Algal clumps were collected haphazardly by either laying down a 7 × 7 cm quadrat and collecting all algae within the quadrat, or by collecting clumps of a particular alga and weighing at the lab. All gastropods were removed by hand under a stereomicroscope, counted, identified to species or morphospecies, measured, and photographed^30^.

### Parasitological collections

We collected fish and invertebrates and dissected them for parasites, with the goal of identifying the most common parasites in the food web. We targeted host groups that are known to transmit trophically-transmitted parasites in other systems. We collected most organisms from mainland sites, and sampled opportunistically at sites on Anacapa, Santa Cruz, and Santa Rosa islands^30^ (Fig. 2). A list of all species dissected and sample sizes is provided^30^.

#### Fish collections

We prioritized collecting the most common and abundant fish species based on survey data from 2000–2014 (SBC LTER), as well as personal observation, expert opinion, and amount of parasite data in the literature. Other species (lower abundance or higher past study effort) were collected opportunistically. Fish were collected primarily by spear on SCUBA. Specific size classes were not targeted and the spear tips used were appropriate for the focal species. Small benthic fish were collected using dip nets. All fish were collected under UCSB IACUC protocol 549.2. Fish were either stored on ice and processed within 24 hours of collection or frozen until processing.

#### Invertebrate collections

Invertebrates are necessary intermediate hosts in many parasite life cycles, but relatively few parasite life cycles have been described in marine environments. We targeted invertebrate species that were abundant and potentially important as intermediate hosts for parasites. We did not collect sessile colonial taxa, such as hydroids, gorgonians, sponges, and tunicates, as they were not expected to be hosts for trophically transmitted parasites (but these hosts do merit further study). Most sampled invertebrates were gastropods and small crustaceans, as they host trophically-transmitted parasites in other food webs. Bivalves, large crustaceans, echinoderms, and polychaetes were also dissected. Large invertebrates were collected by hand or using a rock chisel and scraper when appropriate. Small invertebrates were sampled by collecting benthic substrates in plastic or fine mesh bags and removing organisms in the lab. Invertebrates were held live in flow-through seawater until the time of dissection or frozen until processing.

### Parasitological assessment

For each host dissection, the exterior and all internal soft tissues were examined for parasite life stages. For larger species, entire host organs were usually searched by pressing soft tissues thin between two glass plates (“squashed”) and examining with a stereomicroscope. However, to increase sample size, bilaterally symmetric organs (e.g. gills) were examined from one randomly determined side, and large organs (e.g. muscle, liver) were subsampled in larger fishes. Small crustaceans and soft-bodied invertebrates were squashed whole. We identified gut contents where feasible to improve host diet data and inform parasite life cycles. We recorded host mass, length (or other species-appropriate measurement), collection method, and host condition at time of dissection (e.g. frozen, fresh). We counted and identified all parasites to the lowest possible taxonomic level and assigned a morphospecies code when species-level identification was not possible. Only a few putative parasites were excluded from additional analysis because they had no identifying features. Dissection data^30^ includes species not included in the full food web (see below for discussion of justifications for node inclusion).

### Node list assembly

Nodes in the web included free-living species that used the water column and benthic zones within kelp forests as feeding habitat (including transient kelp-forest visitors but excluding rare and vagrant species) and parasites of those free-living species. Species was the preferred taxonomic unit, and life stages were included as separate nodes if that life stage was present in the system and had distinct trophic interactions from the adult stage. The fully-resolved free-living food web was constructed with life stage (e.g., larva, adult) nested within species (or morpho-species) (excepting benthic diatoms, planktonic diatoms, dinoflagellates, foraminifera, free-living nematodes, bacteria, free-living ciliates, copepod nauplii, filamentous algae, and invertebrate eggs, which were aggregate nodes). As various forms of detritus are important to energy flow in kelp forests, detritus was broken into four categories based on the typical feeding modes of detritivores and main sources of detritus: carrion, drift macroalgae, small mixed origin (such as would be consumed by a deposit or suspension feeder, with the recognition that this alone is a complex system deserving further resolution) and dissolved organic material. The “drift macroalgae” component was especially important to distinguish, as certain herbivores (sea urchins) are known to prefer drift algae as food but will turn to feeding on live algae when drift algae are sparse. This is a very distinct type of interaction from suspension feeders, which consume small particles of detritus that may be largely bacteria. “Parasites” are consumers which fit the seven types of parasitism defined by Lafferty and Kuris^33^. Commensal organisms were also recorded. We limited the parasite species list to metazoan species that use kelp-forest species as hosts for at least one stage in their life cycle. Bacterial, viral, fungal, and protozoan pathogens that are important in kelp-forest food webs merit inclusion in further work.

We assigned each node a justification code (see below), confidence level, literature reference, and locality of the reference. Additional node metadata includes site on host (ecto-vs. endoparasite), taxonomic information, and life cycle information^30^ (see below). The node list contains columns with a species ID, and a species-by-stage ID. To work with the life-stage resolution, select the species-by-stage ID as the node identifier in analyses. To work with the species version, select the species ID as the node identifier in analyses. This will collapse all of the interactions to the species, so all of the trophic interactions are preserved and linked to the species node. Network analysis packages in R (such as Cheddar^34^) will automatically remove duplicate links if they are generated in this process.

#### Life stages as nodes

Species were partitioned into life-stage nodes (e.g., larva, juvenile, adult) if a species changed its trophic position from one stage to the other and multiple stages were present in the system. Whether or not a distinct life stage resided in the kelp forest was indicated by various data sources (e.g. dissections, published records), or inferred from species life history or trophic interactions. For example, amphipods brood offspring and have crawl-away juveniles. These juveniles remain in the kelp forest (rather than having a pelagic phase), and due to their small size are subject to different predators than adults (e.g. adults are eaten by fishes, while juveniles are eaten by hydroids). This was justification for juvenile amphipods being a distinct node from adult amphipods. On the other hand, many species have planktonic larvae that develop outside of the kelp forest, so only the adult stages were included at the species level. Larval stages of parasites were included if there was no feasible alternative for the focal host to become infected. We assumed that kelp-forest resident hosts became infected through life-cycle stages found within the kelp-forest food web, but that transient hosts could have acquired some parasites outside the kelp forest (e.g., if intermediate hosts were not known from the kelp forest). Likewise, presence of larval parasites in dissections was evidence for including adult stages. For some species, there was insufficient data on life history to infer additional stages. Metadata in the node list indicates whether parasites have additional life stages inside the kelp forest, outside, or unknown. When comparing this food web with others (which rarely separate species into life stages), using our data it is easy to collapse life-stage nodes into species nodes.

#### Justifications for node inclusion

We used multiple lines of evidence to justify whether or not to include a node in the food web. Free-living species were included if they were known from the SBC (see site description above) and were indicated by the data sources described above (e.g. reports, surveys, published papers, guidebooks, expert opinion, etc.). Species lists from regional guidebooks included non-kelp-forest species, so these lists were compared with species lists from long-term monitoring surveys. Following the methods of Lafferty *et al*. 2006, we excluded most rare species (<1% frequency of detection in surveys, or those described as “rare” qualitatively). However, we included species that seemed rare because they were cryptic or not looked for, if the species ecological role exceeded abundance (top predators), or if the presence of a final host was inferable based on presence of parasites that require it to complete their life cycle. For instance, a cryptic fish species listed in a guidebook may appear rare in monitoring program surveys, but inclusion might be warranted based on personal observations. For top predators, larval parasites in prey species were evidence for the presence of final-host species (e.g. finding shark tapeworm larvae in a fish indicates a shark is likely present in the system). We also included a few locally extinct or rare species of special conservation or fisheries interest that had a larger historical role (e.g. the sea otter, *Enhyrdra lutris*)^35^ or potential expanded role with global warming. These species are indicated in the node list so they can be excluded or included based on research questions. The justifications for including a node in the food web were included as metadata, as well as the localities of the species observation and references, and then used to determine a categorical confidence score.

Parasites are not as well studied as free-living species, so we used parasite-host records from San Luis Obispo, California to Punta San Hipolito, Baja California, Mexico, corresponding to the dominant biotic province of the SBC. We excluded parasites from outside this range or those known to have freshwater life cycles, as well as ectoparasites of birds. We made exceptions for parasites with additional evidence of presence (such as a larval stage found locally, or a local occurrence in another host species), and for those with transient and wide-ranging hosts^30^. For example, if an adult trematode was observed in pelicans in Florida, but larval stages of this worm had been observed in the Carpinteria (CA) Salt Marsh adjacent to our system, the worm was included. We extended the northern range of acceptable parasite records to San Francisco Bay, California for hosts that were known to migrate between northern and southern California regularly (several species of elasmobranchs, birds, and mammals). This also helped account for the relatively low study effort for these hosts in southern California.

#### Assignment of node confidence

Depending on the evidence for including a node, we rated confidence from 1–4, with 1 being the most confident. Nodes that were observed by monitoring surveys or this study were assigned a confidence value of 1 (61.2% of free-living nodes, 35.5% of parasite nodes). Nodes that were known from the SBC through other sources (e.g. guide books, published literature), but that were not reported in surveys were included with a confidence value of 2 (28% of free-living nodes, 37.7% of parasite nodes). For example, gammarid amphipods were not monitored at the species level in monitoring surveys, but other studies in the region provide lists of local species. Species known from the broader Southern California Bight and with reported ranges north to Point Conception or beyond were included with a confidence value of 3 if they were from a taxonomic group that may not have been sampled effectively by methods utilized in the SBC (6.7% of free-living nodes, 14.4% of parasite nodes). This included several sponge species that were not monitored at the species level by monitoring programs. Transient species indicated by expert opinion and crowd-sourced observations, as well as some life stages that were inferred to be present (e.g. juvenile gammarid amphipod species) were also assigned confidence values of 3. Some parasite life stages that were inferred to be present, but were observed north of Point Conception or outside the greater southern California region were included with a confidence value of 4 (4% of free-living nodes, 12.4% of parasite nodes). We also assigned a confidence level of 4 to parasite nodes whose presence in the kelp forest was less certain due to host transience (large mobile predators that forage across multiple different habitats, not exclusive to kelp forests). Parasites are sometimes mis-identified in published records, so, to avoid false positives, we excluded some parasites on the basis of questionable identifications. These were typically parasites that were only known from one host specimen in one local study but were known from an entirely different group of host organisms in a distant locality. Readers can use confidence scores to filter their own node list.

#### Additional node metadata

Additional metadata for each node includes species functional group (e.g. predator, herbivore, detritivore, omnivore, autotroph, filter-feeder, ectoparasite, etc.), taxonomic information (phylum, class, order, family), habitat association (e.g. holdfast, water column, rock surface, host), small-scale habitat association (e.g. rock, water-column, macroalgae, etc.), geographic range, thermal association, consumer trophic type (Table 1), and consumer strategy (e.g. autotroph, omnivore, detritivore, filter-feeder, carnivore)^30^.

**Table 1:** Types of consumer interactions, following the framework of Lafferty and Kuris 2002.

Trophic Interaction Type	Victim death required?	Victim fitness:	Intensity dependent effect on victim?
***Number of victims (per life stage)*** **>*1***			
Predation	Yes	0	No
Micropredation/grazing	No	>0	Yes/No
***Number of victims (per life stage)*** = ***1***			
Typical parasite	No	>0	Yes
Trophically transmitted parasite	Yes	0	Yes
Parasitic castration	No	0	No
Pathogen infection	No	>0	No

Consumer strategy determined by the number of victims consumed, the fate of the victims consumed, and whether the effect of consumption is intensity dependent. For typical parasites, the effect on the host depends on the number of parasites that infect the host, so such interactions are intensity dependent. Alternatively, a pathogen reproduces within the host, so the effect on the host is the same independent of the number of parasites that initially infect the host.

### Link assignment

Because links in previously published kelp-forest food webs contained errors, we constructed links from scratch using primary sources where possible. Given N nodes in the node list, there are N^2^ potential trophic links (including cannibalism). Many of these potential feeding interactions are easy to exclude based on logic (e.g., giant kelp doesn’t eat animals) and species life history. For example, a subset of free-living species are possible hosts for each life stage and taxonomic group of parasites (e.g. adult tapeworms in the order Trypanorhyncha only infect elasmobranchs). Parasite-host records in the literature are incomplete lists, so we inferred additional links using species life histories and logic. Parasites can also be killed by free-living species when their hosts are eaten (concomitant predation). We used free-living trophic interactions to infer these feeding links between free-living consumer and parasite. Where possible, this food web reports links at the stage level, but these links could be aggregated to the species level, or even the group level for comparison with other food webs. Each link was assigned a literature reference, locality of the observation, justification code, and confidence level^30^.

#### Justifications for link inclusion

Links were assigned using several data sources and logic. A systematic literature review was conducted in Google Scholar™ to collect diet records for each free-living species (including synonyms) using standardized search terms (“Genus species” [diet* OR feed* OR prey]). If these search terms did not yield results, the search was expanded to records of the species (“Genus species”). We also used direct observations from gut contents. In many cases, diet information was not available at the species level, creating the possibility of false negative links (e.g., failing to report a diet item due to lack of direct observation). To reduce the probability of false negative links, the search was expanded to the next higher taxonomic level where information was available, under the assumption that diets are often taxonomically conserved. Such links were inferred by assessing both the compatibility of the interaction (e.g., body size ratios, diet generality), as well as the probability of encounter between the species. For example, if two species were known to encounter each other through shared habitat and behaviors, and general feeding habits of the consumer were compatible with the resource species, a link was inferred. Certain trophic links may only be present seasonally or may vary through time. Temporal data sets provided by the SBC LTER and CINP KFM programs provide abundances over time for many key species in the food web. These data sets could be used to assess temporal stability of links in future studies.

Parasite presence was also used to infer links between free-living consumers and resources when life cycles of parasites were known. The presence of a trophically transmitted parasite in a host indicates that the intermediate host of the parasite was ingested by that host, so a link between those two hosts would be inferred. For some understudied species, expert opinion was used to inform trophic links, so these experts are cited in the links list as the reference for that link. Many experts have unpublished data or observations on feeding interactions and parasite-host interactions, and so were a valuable source of information. We report the strongest justification type for each link in the food web as a justification code, along with all relevant references. For example, if we observed a link directly that was also reported by literature studies, we indicate we used direct observation to justify the link. However, the references for that link indicate it was observed directly and also list the relevant literature, so the reader would know that it was both indicated in the literature and observed directly. For inferences, we list all references that provide the logical basis for an inference (e.g. descriptions of foraging behavior, diet of related species). Justification codes and the numbers of references should not be used as a measure of link weight (e.g. diet proportion), as these often relate more to study effort on the species rather than the importance of the links.

Links between parasites and hosts were assigned using several data sources, as in the free-living web. Direct observations of parasite-host interactions through our sampling or published studies (as detected through systematic review, see “Data Sources” section above) were assigned. However, direct observation of all possible interactions was unfeasible and sampling effort varied among hosts, so parasite-host interactions are often under-sampled. To account for this, links between parasites and hosts were added in stages using the free-living web, host life history, and parasite life history. First, parasite life cycles were inferred based off of known hosts and host trophic interactions. Trophic interactions among free-living species were then used to infer either transmission of parasites to additional hosts or concomitant predation (predator-parasite links) if parasites were not ingested by suitable hosts. Each link is identified by a code that indicates whether it was observed directly (and the source), or whether it was inferred (and the method of inference, described below). Users of the food web can choose to filter links by link justification to suit their needs.

#### Life cycle inference

We used several data sources and considered parasite life histories to assign links with likely hosts. If the life cycle was known for the parasite in another system, we inferred links with analogous hosts in the system (a kelp forest species in the same genus or family). For trophically transmitted parasites, we assessed parasite compatibility with potential hosts, and used free-living trophic interactions to determine whether a parasite would encounter a suitable host. For species with unknown life histories, we considered the life history of the next lowest taxonomic grouping and assumed generalism within that level. For example, the trematode *Podocotyle californica* has an unknown life cycle, but *Podocotyle enophrysi* is known to infect the snail *Lacuna marmorata* as its first intermediate host^36^. Trematodes are host-specific at this stage, and *Lacuna unifasciata* was the only analogous host species in kelp-forest food web, so it was assigned as the most-likely intermediate host for *Podocotyle californica*. On the other hand, marine acanthocephalans are thought to be generalists at the ordinal level in the first intermediate host (D. Marcogliese, pers. comm.) and are trophically transmitted. Although a second intermediate host is not necessarily required for development, acanthocephalans of top predators often use fishes as paratenic hosts. In dissections, fishes were often infected with larval acanthocephalans of birds and mammals, so we assigned amphipod species eaten by infected fish as possible first intermediate hosts. For the 15% of the nodes where a parasite from the dissections could not be identified to family, those without a clear possible host in the kelp forest, or those where nothing was known of the parasite’s life history, we did not make any inferences based on life cycle. Such parasites appear as specialists in the data (but see the false-negative assessment below).

#### Parasite-host inference

The number of parasite species detected is often a function of study effort^37–39^. Because study effort varied among hosts, and was sometimes low, we assigned additional parasite-host links based on potential for encounter with infectious stages of parasites and expected host compatibility. Encounter with trophically transmitted parasites occurs through host diet (i.e. through intermediate hosts eaten as prey) and was informed using the free-living food web and life-cycle inferences as described above. Encounter with directly transmitted parasites occurs through shared habitat or contact with other hosts and was informed by other parasite-host records. We based compatibility on the host-specificity, known hosts in the system, as well as the life stage of the parasite (e.g. adult tapeworms do not survive if their host is eaten, whereas juvenile tapeworms can infect repeated paratenic hosts and remain viable). For example, if a monogene was reported from 15 rockfish species in British Columbia and observed in two species locally, it was assumed to infect other rockfish species present in the SBC kelp-forest food web.

#### Predator-parasite interactions

Host death by predation is a major source of parasite mortality and may strongly influence parasite-host dynamics. We inferred these predator-parasite interactions using trophic interactions between free-living species. For each free-living consumer interaction, we assessed whether the parasites of the prey host would be killed or transmitted to the predator. If the predator was not a compatible host (see discussion above), we assigned a consumptive link between the free-living consumer and parasite. Users should consider that although predator-parasite links influence parasite vulnerability, they rarely constitute a significant flow of energy from parasites to predators. Food-web metrics that imply energy transfer (e.,g robustness and other bottom-up effects) should therefore not include predator-parasite links.

#### Assignment of link confidence

Although inferring links from logic reduces the frequency of false negative links, it also increases the possibility of reporting false positive links (reporting links that do not in fact occur). To help indicate confidence, links were assigned a code from 1–4 based on the strength of the justification for the link, with 1 being the most confident, 4 being the least. Links from the literature were assigned a confidence code based on the proximity between SBC and the region where the interaction was observed. Any links indicated by direct observations, or other studies conducted within the SBC were assigned a confidence value of 1. Links indicated by literature conducted within the greater southern California region were assigned a confidence level of 2, if the links were species-specific. Species-specific links in the literature that were from outside southern CA were assigned a confidence value of 3. Some non-species-specific links from within the SBC or southern CA were also assigned a confidence value of 3 if there was evidence that the species involved matched those in this web. Links that were inferred from only a single line of indirect evidence, or those that lacked locality or reference information were assigned a confidence level of 4. When inferred host-parasite links were based on information from inferred predator-prey links, confidence values were set to the lowest confidence value of the information that led to the inference. For example, if an adult trematode infected kelp rockfish with confidence level 3, and leopard sharks ate kelp rockfish with confidence level 2, a concomitant mortality link (predator-parasite) was assigned between the leopard shark and the trematode with confidence level 3. Therefore, the confidence score should correlate inversely with the probability that a proposed link is a false positive and indicates where more study is needed. A caveat is that the confidence values are qualitative as they represent categories, rather than a quantitative spectrum of certainty, so some uses of the confidence values (such as assigning averages or variances) would not necessarily represent a sound use of the data. Confidence values should also not be used to indicate strength or weight of a link, as they only indicate confidence in the presence of the link.

#### False negative estimation for host-parasite links

Even though many unobserved host-parasite links were inferred to occur based on logic, under-sampling leads to the potential for other false negative links. Such links are particularly likely for generalist parasites that have low prevalence in under-sampled hosts. For instance, if a metacercaria species infects any rockfish species at 5% prevalence, and we sample ten individuals from each of ten rockfish species, we can expect by chance to observe the parasite in only six of the ten species. The remaining four rockfish species might appear to be uninfectable by the parasite, but, assigning 0 s in the bipartite host-parasite network would result in false negative links. False negative links make parasites look more like specialists than they actually are, thereby underestimating their importance in food-web measures such as generality, vulnerability, linkage density, and connectance (the proportion of realized links relative to the number of possible links). We estimated false-negative probabilities for unobserved links at the species level and individual host level (we assumed the probability of a false positive observation was low enough to be ignored unless noted). We applied this approach separately to the following bipartite networks: trophically transmitted parasite-fish, directly transmitted parasite-fish, parasite-shark, parasite-bird, and parasite-mammal.

The first step to estimating a false negative probability is to calculate a prior statistical expectation that a parasite group infects a host group based on previously reported host-parasite links in the literature. At the node-level, we used a generalized linear model with observed or inferred link (0,1) as a dependent variable and taxonomic information (host order, host family, parasite order, parasite family, parasite species), host trophic level (calculated from the free-living web), host habitat association, and proportion of the host diet that may contain infective stages as independent variables (JMP Pro V14^40^). Because false negatives arising from under-sampling are common in the parasitological literature^39^, we included a square-root transformed sampling effort term (the number of parasite studies on the host in the literature). Model selection was based on Akaike information criterion (AIC)^41^, and found that host and parasite taxonomy and traits helped predict links (see Table 2 for model results of each network). The interaction between host order and parasite family was important in all bipartite networks, indicating parasite specialization at higher taxonomic levels. Study effort was less important in subnetworks with higher sampling effort across hosts. From the best-fitting model, we generated predicted probabilities for each link between species *i* and *j*, at existing effort ${\widehat{\psi }}_{ij}$. We then assumed that with increasing effort, the probability that a link was observed ${\widehat{\psi }}_{ij}$ approached the probability that the link exists *Ψ*_*ij*_. Then, by parameterizing the prediction equation with a hypothetical “high” effort (see Table 2) for values for each bipartite network), we projected the probability that a link exists ${\widehat{\varPsi }}_{ij}$. According to Bayes’ Theorem, the probability of a false negative *F*_*ij*_, is:

$${\mathbb{P}}({\varPsi }_{ij}=1\,\& \,{\psi }_{ij}=0)/{\mathbb{P}}({\psi }_{ij}=0)$$

Which translates to:

$${F}_{ij}=({\widehat{\varPsi }}_{ij}-{\widehat{\psi }}_{ij})/(1-{\widehat{\psi }}_{ij})$$

Which is a first approximation for the probability of a false negative link based on species-level data. Namely, the more likely a link occurs based on taxonomy and traits, and the less likely it is to be sampled with existing effort, the more likely an unobserved link is a false negative link due to insufficient sampling effort. We therefore estimated ${\widehat{\varPsi }}_{ij}$ (and its standard error) and ${\widehat{F}}_{ij}$ from data at the species level.

**Table 2:** Generalized linear models used in false negative estimation.

	Mammals				Birds				Sharks				Fish - Trophic Transmission				Fish - Direct Transmission			
	*Nparm*	*df*	*Wald χ*^2^	*p* > *χ*^2^	*Nparm*	*df*	*Wald χ*^2^	*p* > *χ*^2^	*Nparm*	*df*	*Wald χ*^2^	*p* > *χ*^2^	*Nparm*	*df*	*Wald χ*^2^	*p* > *χ*^2^	*Nparm*	*df*	*Wald χ*^2^	*p* > *χ*^2^
Host Order x Parasite Family	14	4	39.46	<0.0001	44	27	435.9	<0.0001	210	59	463.98	<0.0001	301	74	846.33	<0.0001	252	54	320.85	<0.0001
Host Family [Host Order]													17	14	325.96	<0.0001	18	12	322.91	<0.0001
Host Order	1	1	15.05	0.0001	4	3	37.56	<0.0001	6	2	11.32	0.0035	7	3	231.76	<0.0001	6	2	145.09	<0.0001
Parasite Family	14	5	31.89	<0.0001	11	11	176.832	<0.0001	35	13	11.53	0.567	43	11	221.6	<0.0001	42	4	93.61	<0.0001
Host Habitat													5	5	165.39	<0.0001	6	5	58.13	<0.0001
Parasite Node [Parasite Family]					14	7	131.3	<0.0001					105	42	246.15	<0.0001	117	26	213.53	<0.0001
Host Trophic Level									1	1	12.99	0.0003	1	1	101.73	<0.0001	1	1	38.31	<0.0001
Prop. of diet that could transmit parasite													1	1	15.86	<0.0001				
√(Study Effort)	1	1	2.21	0.1369	1	1	14.46	0.0001	1	1	5.7	0.017	1	1	10.45	0.0012	1	1	72.79	<0.0001
AICc	106.1				297				538.7				2793				2625			
R^2^	0.516				0.593				0.504				0.346				0.247			
N rows	87				442				710				10132				10720			
Hypoth. max effort	75				40				10				10				10			

Separate models were constructed for each of the following bipartite networks: parasite-mammal, parasite-bird, parasite-shark, trophically transmitted parasite-fish, and directly transmitted parasite-fish.

We also had individual-level data for many potential links, making it possible to refine the estimate for ${\widehat{F}}_{ij}$ based on dissections. Now, Bayes’ Theorem translates to:

$${\widehat{F}}_{ij}={\widehat{\varPsi }}_{ij}(1-{\widehat{d}}_{ij})/(1-{\widehat{d}}_{ij}\,{\widehat{\varPsi }}_{ij})$$

Where ${\widehat{\varPsi }}_{ij}$ is estimated as above from the prior species-level data and is ${\widehat{d}}_{ij}$ link detectability from dissections (the probability of detecting a link in a sample if that link occurs). ${\widehat{d}}_{ij}$ can be estimated from individual-level data (e.g., several dissected host individuals). In a host species *j* that is known to be infected by a parasite species *i*, the probability *d*_*ij*_ of finding an infected individual after dissecting *K* hosts is akin to a series of *K* independent Bernoulli trials, each with a probability of detecting a parasite in a host equal to the parasite’s prevalence in the host population, *p*_*ij*_.

$${\widehat{d}}_{ij}=1-{(1-{p}_{ij})}^{{{\rm{K}}}_{j}}$$

In the case of a host species where a parasite species *i* has never been detected, the parasite’s detectability in dissections is also akin to a series of *K* independent Bernoulli trials, but the parasite’s prevalence in the host population must be estimated from infectable hosts. The simplest assumption is that infectable species do not differ in prevalence, so that ${\widehat{p}}_{ij}$ is just the number of individual (*h*) parasitized hosts $\left({\sum }_{\left(h=1,j=1\right)}^{\left(h=K,j=m\right)}{\psi }_{hij}\right)$ found in combined samples from those *m* host species that are infectable by parasite species *i*. E.g.,

$${p}_{ij}=\frac{{\varPsi }_{ij}{\sum }_{\left(h=1,j=1\right)}^{\left(h=K,j=m\right)}{\psi }_{hij}}{{\sum }_{j=1}^{m}{K}_{ij}}$$

Which we estimated as

$${\widehat{p}}_{ij}=\frac{{\psi }_{ij}{\sum }_{\left(h=1,j=1\right)}^{\left(h=K,j=m\right)}{\psi }_{hij}}{{\sum }_{j=1}^{m}{K}_{ij}}$$

Although there are more complicated ways to estimate prevalence that take into account individual host traits, and biases from excluding infectable hosts where infections have not been detected, the simple method was sufficient to distinguish between likely and unlikely false negatives. Thus, to recap, we estimated ${\widehat{\varPsi }}_{ij}$ using species-level data as above, then further refined the estimate of ${\widehat{F}}_{ij}$ from dissection data. We used error propagation to report 95% confidence limits^30^.

With information about ${\widehat{F}}_{ij}$, we estimated unseen parasite-host links as probabilities, rather than as 0 s (observed links were set to 1, and unobserved links were set to ${\widehat{F}}_{ij}$). Doing so identified some likely parasite links that were missed. In this case, when the probability of a false negative was >0.5, we assumed that an unobserved link actually occurred unless otherwise contradicted by species life history. We also then noted the probability of a false positive link (1 - ${\widehat{F}}_{{\rm{ij}}}$). We further identified those few host and parasite species that generated substantial error in the network. To keep the overall error rate to <4%, we therefore removed error-prone species from the network^30^. These species were typically rare generalists that were easily missed in dissections. To that extent, the decision to remove them was consistent with our decision to remove rare free-living species from the network. We report these removed species and their known links^30^ as potentially useful information for other purposes. Finally, we used the false-negative estimates to correct for biases in network and species-level measures like generality, connectance, and linkage density.

#### Additional link metadata

In addition to metadata on locality, literature source, justification, and confidence, we categorize links based on different types of trophic interactions. We specified the interaction type for each consumer-resource link following the framework of Lafferty and Kuris^33^ (Table 1). For instance, links where a consumer kills the resource were coded as predator-prey interactions, while links where a consumer eats a small portion of a resource individual without killing it (e.g. herbivores) were assigned as micropredator/grazer interactions. Thus, the free-living web contained predation and micropredation/grazing links. Some organisms often referred to as “parasites” fit the definition of micropredation (e.g. gnathiid isopods). Several more types of interactions are possible between symbiotic organisms and their hosts, depending on transmission strategy (trophic transmission or direct transmission), effects on host fitness, and reproduction method (within the host or in the environment). Metadata in the node list (such as site of infection) allows investigators to simplify these link types according to research questions of interest.

## Data Records

The data package includes 14 files (.csv), four directories containing several thousand images of organisms, and metadata for the directories^30^. Each data file is described in Table 3. The food web itself is comprised of a nodes list (“1_Nodes.csv”) and links list (“2_Links.csv”). References for these two lists are contained in “13_Ref.ID.csv”. The remaining files provide detailed dissection data, free-living organism sampling data, and information on false negative links (Table 3). The image directories contain photos of parasites, as well as the free-living organisms found in zooplankton sampling, holdfast sampling, and small gastropod collections. “READ ME” documents included within each directory describe the image file naming system and which data file the images correspond to.

**Table 3:** Description of data files included in the data package.

File Name	Description
0_Column_Descriptors.csv	Column descriptions for all data files.
1_Nodes.csv	Nodes list for the food web, including node metadata.
2_Links.csv	Links list for food web, including link metadata.
3_Zooplankton_data.csv	Zooplankton sampling data, associated photos in Zooplankton_Photos_Kelp_Forest.tgz
4_Holdfast_data.csv	Holdfast sampling data, associated photos in Holdfast_Photos_Kelp_Forest.tgz
5_Small_Gastropods.csv	Small gastropod sampling data, associated photos in Small_Gastropod_Photos_Kelp_Forest.tgz
6_Sampling_sites.csv	Sites where sampling for parasites occurred.
7_N_Dissected.csv	Species dissected and sample sizes. Some species dissected were not included in the food web if they did not meet abundance criteria but are included here as they may be useful for other parasitological studies.
8_Dissection_data.csv	Dissection data for fish and invertebrate hosts. Some species did not meet criteria for inclusion in the final food web but are included here as they may be useful for other studies. Associated photos in Parasite_Photos_Kelp_Forest.tgz
9_Record_range_expansions.csv	Host species for which parasite records were included from San Francisco Bay, CA, USA to Punta San Hipolito, Baja California, Mexico.
10_FN_probabilities.csv	Probabilities of false negative links for each possible link in the following bipartite networks: parasite-mammal, parasite-bird, parasite-shark, trophically transmitted parasite-fish, and directly transmitted parasite-fish.
11_Species_removed_FNs.csv	Parasite species removed from network due to high error in false negative predictions.
12_Links_removed_FNs.csv	Links removed due to high false negative errors.
13_Ref.ID.csv	References and corresponding numeric codes used in 1_Nodes.csv and 2_Links.csv

Data available from the Dryad Digital Repository^30^: 10.25349/D9JG70.

## Technical Validation

Multiple lines of evidence were used to justify node and link inclusion. References are included for all nodes, body size estimates, and links. Where links were inferred, the references which provided the logical basis for the inference are listed, along with a code indicating the specific type of inference (e.g. “closely related species in literature”).

## Usage Notes

We organized both the nodes and links data and metadata to facilitate analyses and appropriate usage by other researchers. The food web is resolved to life stage and includes free-living species and parasites, but nodes can be aggregated or separated by taxonomy, lifestyle, or habitat niche. The links list can be filtered by trophic interaction type, justification, or confidence. Code definitions for justification, confidence, and trophic interaction type are described in metadata^30^. Correct interpretation of food-web structure requires understanding limitations, which are often dissociated from food web data sets in repositories. We describe the limitations of the food web database below.

### Food-web boundaries

Food-web research in general may limited by “soft boundaries” of the food web, as very few food webs are completely isolated from their surroundings. We restricted our definition of kelp forests to rocky-reef habitat and the water-column above it, but kelp forests can have sand channels throughout and are often surrounded by sand. For this reason, we included sand-dwelling species that are known to associate with kelp forests specifically, however we did not include the sand community in general, even though this habitat is often interspersed and adjacent to the kelp forest. Once a subtidal sandy food web has been created, it should be straightforward to connect kelp forest and sand-associated food webs.

### Life stages as nodes

Although the food web separates distinct life stages into separate nodes, it does not include multiple size classes for each species. Changes in diet associated with size are common across fishes and could alter network structure. Additional resolution could be added to the web by including size classes for species that experience strong ontogenetic shifts in diet.

### Species abundances

Because our web is a meta-web for a larger region, we did not include density estimates for species. However, site-specific densities are available for >200 organisms surveyed by CINP KFM (https://irma.nps.gov/DataStore/SavedSearch/Profile/1508) and the SBC LTER program ongoing community timeseries (https://sbclter.msi.ucsb.edu/data/catalog/). Adding this information, and perhaps inferring densities for other taxa based on allometric scaling might make it possible to use this food web for dynamic modeling.

### Node inclusion

Although this food web improves resolution for many groups of organisms (including crustaceans, gastropods, invertebrates, birds, cryptic fishes), it does not capture all species or links. This is a commonly cited criticism of food webs, in particular large networks (e.g.^42–46^). We attempted to minimize this by using information from many sources, inferring links, and constructing a web that was cumulative over space and time. We did not attempt to resolve other potentially important taxa like protozoa (ciliates, flagellates, etc.), diatoms, and other microbes (viruses, bacteria, fungi). The food-web construction allows for additional types of organisms, life stages, and interactions to be added. Nodes such as small particles of detritus represent their own complex systems that surely deserve future study. Although more sampling would lead to a longer and more complete species list, new additions would more likely be uncommon species that contribute less to biomass and energy flow than do the species included here. Additional sampling would be expected to further increase network size and complexity, but adding or removing small numbers of species did not affect overall network structure.

### Link inclusion and weighting

This food web does not include interaction strengths (link weights). In many cases, adaptation is realized in terms of changes in link weights, so topological food webs in general are limited in their ability to detect adaptation to change. Adding link weights to a network of this size would be very complex, and potentially impossible, because interaction strengths may vary seasonally, spatially, and temporally.

The near future will likely see enhanced molecular approaches in food-web research as barcoding and eDNA-based techniques continue to develop. This could allow for inclusion of additional cryptic and transient species, as well as statistical inference of species associations (co-occurrence). At present these techniques would only be possible for a small subset of well-known species (sharks, some fish, marine mammals), as many invertebrates and parasites do not have reliable species-level sequences, but these methods may be useful for detecting top predator presence^47^.

In addition to missing nodes, there are likely numerous missing (false-negative) links. We focused on missing links between existing nodes, but missing links also occur between existing and missing nodes, and between missing nodes. We attempted to correct for false negative host-parasite links through inference of parasite life stages, additional host interactions, and false negative estimation, but recognize that additional sampling and resolution of cryptic diversity would improve network accuracy. Although missing links bias food-web properties, by estimating false-negative probabilities, it is possible to correct for much of this bias simply by replacing 0 s with false negative probabilities when computing network statistics that count observed links.

Although false negatives are a concern in ecological networks, false positives are possible due to inaccurate life cycle inferences, particularly for parasites with assumed low-host specificity. Our assumptions of parasite generalism were supported by literature and expert opinion (Marcogliese pers. comm.,^48,49^). By assuming generalism at the level indicated by parasite life history, we ensured that at least one correct host (likely more) was included, with reduced chance of false negatives. Parasite species for which generalism in larval stages was assumed (a few nematodes, some tapeworms, and acanthocephalans) were widespread in many second-intermediate and paratenic host species in dissections, suggesting that there should be more than one infection pathway for such a wide range of hosts to become infected. However, use of paratenic hosts makes it more challenging to identify first intermediate hosts by diet alone. We restricted assumptions of generalism to cosmopolitan parasites of wide-ranging hosts that may be less likely to host cryptic species due to increased gene-flow among populations^50^. By including link justification and confidence levels, readers can treat these links as predictions and filter the node and links list to suit their research questions. Despite these limitations, we note that few other studies justify reported food-web links or distinguish between inferred and observed links. The metadata included with the links list makes users of our data aware of limitations and will ensure that conclusions drawn from the food-web are appropriate to the data.

## Data Availability

Generalized linear models used in false negative estimation were conducted in JMP Pro V14^40^. Network figures were created with the *igraph* package in R Version 3.6.2^51^.
